# Behavioural thermal regulation explains pedestrian path choices in hot urban environments

**DOI:** 10.1038/s41598-022-06383-5

**Published:** 2022-02-14

**Authors:** Valentin R. Melnikov, Georgios I. Christopoulos, Valeria V. Krzhizhanovskaya, Michael H. Lees, Peter M. A. Sloot

**Affiliations:** 1 Complexity Institute, Nanyang Technological University, Singapore, Singapore; 2 Future Cities Laboratory, Singapore-ETH Centre, Singapore, Singapore; 3 Decision, Environmental and Organizational Neuroscience Lab, Nanyang Business School, Nanyang Technological University, Singapore, Singapore; 4 Informatics Institute, University of Amsterdam, Amsterdam, The Netherlands; 5 National Centre for Cognitive Research, ITMO University, Saint Petersburg, Russia; 6 Institute of Advanced Study, University of Amsterdam, Amsterdam, The Netherlands; 7 Complexity Science Hub, Vienna, Austria

**Keywords:** Neuroscience, Computational models, Environmental impact, Climate-change adaptation, Psychology and behaviour

## Abstract

Due to phenomena such as urban heat islands, outdoor thermal comfort of the cities’ residents emerges as a growing concern. A major challenge for mega-cities in changing climate is the design of urban spaces that ensure and promote pedestrian thermal comfort. Understanding pedestrian behavioural adaptation to urban thermal environments is critically important to attain this goal. Current research in pedestrian behaviour lacks controlled experimentation, which limits the quantitative modelling of such complex behaviour. Combining well-controlled experiments with human participants and computational methods inspired by behavioural ecology and decision theory, we examine the effect of sun exposure on route choice in a tropical city. We find that the distance walked in the shade is discounted by a factor of 0.86 compared to the distance walked in the sun, and that shadows cast by buildings have a stronger effect than trees. The discounting effect is mathematically formalised and thus allows quantification of the behaviour that can be used in understanding pedestrian behaviour in changing urban climates. The results highlight the importance of assessment of climate through human responses to it and point the way forward to explore scenarios to mitigate pedestrian heat stress.

## Introduction

The global process of climate change poses a significant threat to urban populations. The population growth, happening mostly in the cities^1^—the areas mainly contributing to the climate change^2^ and strongly affected by higher temperatures^3^—results into an increasing number of people being exposed to excessive heat. This in turn challenges many aspects of modern society: public health^4,5^, human-^6^ and economic development^7^, mental health^8^ and social relations^9^. It is therefore critically important to understand the ways to improve urban thermal environments and the human response to these improvements.

Modelling urban climate at the pedestrian level allows to predict the thermal environment^10–13^. Thermal perception and acceptance studies conducted through surveys throughout the world^14–17^ connect the microclimate and comfort of the people in it. These developments allow to evaluate and introduce the design and planning measures to improve pedestrian thermal environments through green^18–20^ and built^21,22^ shading infrastructure, orientation of buildings^23^ as well as smart path planning^24,25^. Yet, to evaluate the implication of these design and planning solutions for urban residents, it is necessary to gain a quantitative understanding of pedestrian behavioural responses to varying urban microclimates.

Attendance of urban areas and occupation of sun and shade^26,27^, duration and intensity of activities in urban parks^28^ and preference for a sun-lit side of a street^29^ have all been found to have an association with climate. While these rather qualitative observations confirm the presence of a pronounced human behavioural response to thermal environments, they do not provide insights into mechanisms underlying such behaviour, which are crucial for development of the quantitative model of pedestrian behavioural response to urban thermal environments.

Research on crowd dynamics^30,31^ and human navigation in urban environments^32^ provides mechanistic models of pedestrian motion, which are able to reproduce the aggregate dynamics or distribution of pedestrian flows in the environment. These proposed mechanisms, however, remain hypothetical, requiring formal testing of them through controlled experimentation and quantitative characterisation of pedestrian choices in natural environments. Current literature lacks such studies, owing primarily to complexity of the problem of pedestrian decision making in complex urban environments.

Choice modelling has been successfully employed to uncover the decision processes in many domains—from finance and behavioural economics to transportation, marketing, food preferences and animal behaviour^33–36^. In such studies, participants make binary choices that force them to evaluate the effect of, usually antithetical, decision parameters. Critically, this so-called two-alternative forced choice (2AFC) methodology allows for the development and testing of increasingly precise models that parametrically study and predict behaviour at the individual and aggregate level. Requiring exact characterisation of the parameters of the choices such studies are usually constrained to controlled laboratory environments and have not yet been applied to study pedestrian behaviour in urban spaces.

In this study, we combine experimental methods and computational approaches, inspired by behavioural ecology and decision theory, to examine the hypothesis that pedestrians adapt their path choice in order to reduce their sun exposure in a tropical city (Singapore). We examine the interactive effects of two key path choice parameters: distance and shading. In our experiment, participants were asked to perform a series of binary path choices in a natural outdoor environment in Singapore. We use a computational model of the experimental area to precisely parameterise sun exposure in path choices faced by participants. We formulate a hierarchical probabilistic model of choices and infer the values for the perceived cost of walking under the sun, both at the individual and population level. The effect of sun avoidance is mathematically formalised, providing a quantification of the behaviour that can be used in simulations and other forms of quantitative analysis. Our work demonstrates how traditionally in-lab behaviour research methods, being supported by computational tools, can be applied in dynamic settings to study human behaviour in naturalistic environments. We confirm that pedestrians actively avoid sun through path choice behavioural adaptation, which has direct implications for the urban design and policy in changing climate.

## Results

In this study we formulated the model of pedestrian path choice behaviour driven by the length and sun exposure parameters of the path options. To do so we designed the controlled experiment with human participants following the two-alternative forced choice methodology. The design of the experiment and the dataset resulting from it is reported in the following subsection. We then demonstrate how the parameters of path options and of participant preferences influence the interpretation of the choices they made. Finally, we provide the complete model of path choices and the results of estimation of its individual- and population-level parameters.

### Human behaviour experiment and resulting dataset

**Figure 1:**
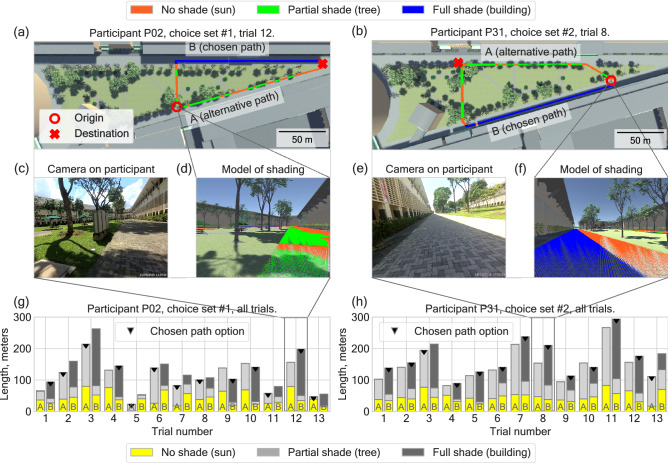
Description of the path choice behaviour experiment and resulting dataset. **(a,b)** At each trial participants of experiment were tasked to reach the destination indicated by cross by walking on one of two paths specified on schematic map of the experimental area given to them. Depending on the time of the year the sun was positioned in either the northern **(a)** or southern **(b)** part of the sky. To maximise the number of choices with a trade-off between sun exposure and distance, two different choice sets for two times of the year were designed. **(c,e)** Participants wore action cameras to record their actions and decisions, which were later used to reconstruct the decisions and to calibrate the 3D model. **(d,f)** A calibrated computational model of the experimental area was used to calculate the sun-shade composition of the path options. **(g,h)** Each participant completed a series of 13 trials, each being a choice between two path options characterised by sun-lit (yellow), tree-shaded (light grey) and building-shaded (dark grey) length. The figure is generated with use of package matplotlib v3.2.2 (https://matplotlib.org/) for Python v3.7.7 and Unity 3D (https://unity.com/) v2019.2.19f1.

The experiments have been performed in the courtyard of the National Institute of Education, at the campus of Nanyang Technological University in Singapore during the period from June to December 2019. The experimental area is characterized by two wide walking paths next to buildings which frame a triangular shaped lawn area. Multiple paths cross the lawn area, connecting the two wide paths. Depending on the time of the year, one of the paths is exposed to the sun, whereas the other is shaded by buildings. Based on this, two choice sets for participants were designed: choice set #1 for the period of June–October 2019, when the northern path was shaded by the building and the southern path was exposed to the sun (Fig. 1a). Choice set #2 was designed for the period November–December 2019, when the southern path was shaded by the building and the northern path was exposed to the sun (Fig. 1b). As Singapore is situated close to the equator and is characterized by a stable hot and humid climate, we assume that there was no significant impact of seasonal variation of climate (apart from the sun position) on the experimental procedures and outcomes.

In each trial, participants were asked to move to a target in the area by taking one of the two paths specified on a schematic map (see Appendix A for the experimental booklets given to participants). Upon reaching the target, the participant proceeded to the next trial with a new target (for the full description of experimental procedure see “Methods”). Figure 1c,e show still captures obtained from video cameras (mounted on each participant—see “Methods”). The video data from each participant is used to identify the path chosen in each trial. A 3D virtual reconstruction of sun exposure was implemented to facilitate precise and reproducible parameterisation of the choices of participants (see Fig. 1d,f). Figure 1c shows video image from participant P02 at the origin of trial 12 and Fig. 1d illustrate the same position in the 3D virtual reconstruction. Figure 1e,f show the same for trial 8 of participant P31. The detailed 3D reconstruction made it possible to estimate the exact composition (in terms of sun-lit [orange], tree-shaded [green] and building-shaded [blue] fractions) of each path option provided to each participant at the moment of decision (Fig. 1a–f). The details of the model-based estimation of these parameters can be found in the “Methods” section. Each participant completed 13 trials in total (Fig. 1g,h), of which one was a test trial: providing a choice between a significantly longer sunny path and a shorter, less sunny, alternative. Figure 1g,h includes information about the length of the path options for each trial. This length is further broken down into shaded (tree and building) and sun-exposed sections.

Path options are labeled *A* and *B*, where option *B* denotes the path with generally more building-shade. Depending on the season (i.e. location of building shade), path option going through either northern or southern side of the experimental area is labelled as *B*. Path options were not labelled with letters in experimental booklets given to participants, and shading patterns were not visualised in these booklets (see Appendix A).

In total 74 individuals from the university students, staff and visitors took part in the experiment. 18 participants were not included in the analysis for various methodological reasons (missing data, rainy weather, failed test trials, etc.). Of 56 participants considered eligible for the analysis in this study, 46 (15 female) participants had made at least one decision in the presence of sun (treatment decisions). In total 408 treatment decisions of these 46 participants are analysed in this study.

This study was preregistered prior to the analysis of the data (available at https://osf.io/q5hnk/). Details of the data processing are described in the "Methods" section.

### Available path choice strategies are determined by the parameters of path options

**Figure 2:**
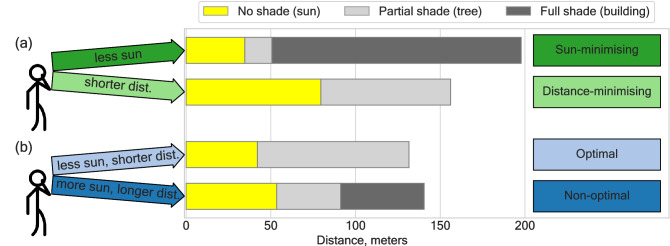
Parameters of path options define possible path choice strategies. Depending on the environmental conditions and the spatial configuration of path options in a particular trial, participants faced a choice either between less sunny but longer (sun-minimising) vs. sunnier but shorter (distance-minimising) options **(a)**; a second type of choice was between less sunny and shorter (optimal) vs. sunnier and longer (non-optimal) options **(b)**. The choices between sun-minimising and distance-minimising options **(a)** allow us to quantify the trade-off between sun exposure and walking distance in the process of pedestrian path choice behaviour. The figure is generated with use of package matplotlib v3.2.2 (https://matplotlib.org/) for Python v3.7.7.

To model the choices of the participants and the decisions made by them we consider two parameters of the path options provided to participants in this study: exposure to the sun and walking distance. Following similar approaches from behavioural economics and ecology (where, for instance, a “smaller [in money] and sooner [delivered in time]” option vs. a “large [in money] and longer [in time]”), the choice sets (points of origin and destination, path options) were designed in a way to allow choices between sunnier and shorter vs. less sunny but longer path options (Fig. 2a). Choosing the former constitutes a distance-minimising strategy of the participant, while choosing the latter corresponds to a sun-minimising strategy. The choices between these two alternatives allow us to quantify the trade-off between sun exposure and walking distance in pedestrian path choice behaviour. We model this trade-off by a participant-specific parameter of perceived cost of walking under the sun $\beta _j > 0$. The cost of walking along a particular path option is defined as the length of this path, sun-exposed portion of which is weighted by the parameter $\beta _j$. For values of $\beta _j > 1$ the cost is inflated reflecting depreciation of sun exposure. This leads to possibility of higher cost of walking associated with shorter but more sun-exposed path option. The primary goal of this study is to estimate this parameter on individual and population level to quantify people’s preferences and path choice behaviour in hot urban environments.

As the experiment is carried out in a semi-naturalistic environment—in order to increase the ecological validity while maintaining experimental control—the position of the sun in combination with the space configuration allowed the emergence of another type of choice. In such cases, participants were facing a choice between less sunny and shorter vs. sunnier and longer path options (Fig. 2b). Choosing the former constitutes an “optimal” strategy by the participant (minimising both exposure to the sun and walking distance), while choosing the latter corresponds to a “non-optimal” strategy, contradicting our assumption of the rational pedestrian, who minimises either sun exposure, or walking distance, or both.

### Tree shade is perceived differently from building shade

While classifying the choices in Fig. 2, we only consider the length of the path exposed to sun and the total length of the path, thereby assuming that tree shade is identical to building shade. While this is necessary to explain the classification of the decisions, this assumption is rather simplistic as tree shade might be perceived differently as compared to the shade of a building. We define perceived tree shade intensity as the amount of shading relief participants associate with tree shade as compared to building shade. A 100% tree shade intensity would correspond to tree shade being perceived as identical to building shade, whereas 0% would mean that participants consider tree shade as identical to full sun exposure. In terms of discounting and path choice we assume that a given length of partial (tree) shade can be decomposed into equivalent full sun and full (building) shade lengths. For example, a 50% tree shade intensity over 100 m is equivalent to 50 m of full sun with 50 m of full (building) shade. To numerically represent the concept of perceived tree shade intensity, we integrate the parameter $\rho \in [0, 1]$ into the path choice model. In Appendix C we demonstrate how various values of this parameter affect the classification of participants’ choices. As perceived tree shade intensity parameter decreases, the choices previously classified as non-optimal get classified as sun-minimising, suggesting that this parameter plays a critical role in correct interpretation of participant decisions. Estimation of the perceived tree shade intensity parameter of human path choice behaviour has important implications for the planning of urban areas.

### Modelling path choices reveals perceived cost of walking under the sun

We formulate probabilistic model of path choices in our experiment. In this model probability of choosing one of two path options is driven by the difference between costs of walking associated with each option. The cost of walking in turn depends on the sun-shade composition of the path option, which is obtained from the validated 3D model of experimental area (see “Methods”), and two model parameters: participant-specific distance-inflating coefficient (cost factor) of walking under the sun $\beta _j$ and perceived tree shade intensity $\rho$ (see “Methods” for the exhaustive model formulation).

We estimate model parameters using 408 precisely characterised (in terms of sun-shade composition of path options) path choices made by participants of our experiment. We assume that the decisions are made independently of each other, based on the sun-shade composition of the currently presented decision options. While this assumption neglects the possible effect of order, fatigue or accumulated heat stress on decisions, it appears reasonable for the duration of experiment (20–30 min). This translates into an assumption of static parameters of the participant decision model ($\beta _j$). The number of data points (maximum 13 per participant) limits the complexity of the model we can apply.

We estimate the parameters in a Bayesian framework. We define a hierarchical model: we set the hyperprior distribution for the parameters of the prior distribution of $\beta _j$. This allows the distance-inflating coefficient of the sun to be estimated for each individual participant, while still being constrained by the overall distribution observed at the population level^37^. We use the PyMC3^38^ implementation of Markov Chain Monte Carlo for parameter estimation. The full specification of the Bayesian model and estimation procedure are described in the “Methods” section.

**Figure 3:**
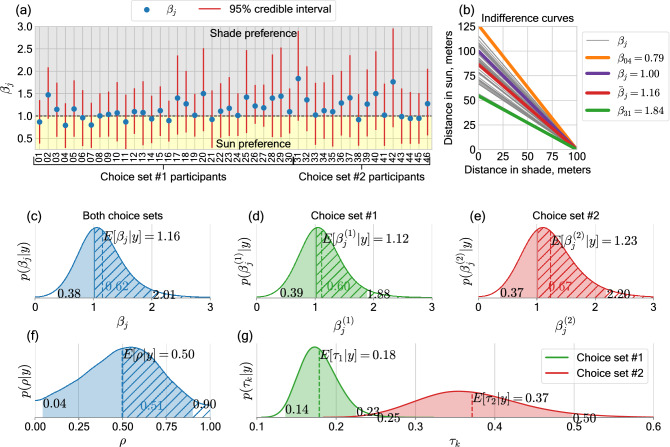
Estimated parameters of the hierarchical model of path choices. **(a)** The expected value and 95% credible interval of participant-specific distance-inflating coefficient of the sun $\beta _j$. $\beta _j > 1$ means preference towards shade in the process of path choice. **(b)** The distance inflating coefficient of the sun regulates the slope of the curve of indifference between distance walked in sun and distance walked in shade. Indifference curves of individual participants are shown in grey. $\beta _j = 1$ corresponds to complete indifference, whereas $\bar{\beta }_j=1.16$ corresponds to the average value of the coefficient estimated from the path choices of all participants. On average participants were indifferent between walking 100 m in full shade, 93 m of mixed exposure (50 m in full shade + 43 m in the sun) and 86 m in the sun. **(c)** The posterior distribution of the distance-inflating coefficient of the sun for path choices pooled for all participants, participants with choice set #1 **(d)** and choice set #2 **(e)**. 95% credible intervals are depicted as filled regions. Hatched regions correspond to the mass of the posterior distribution over $\beta _j>1$. **(f)** The posterior distribution of the perceived tree shade intensity $\rho$; the expected value of 0.50 implies that, on average, tree shade is perceived as 50% as intense as the building shade. **(g)** Posterior distributions of cost-difference scaling parameters $\tau _k$. The disjoint 95% intervals of the two distributions can be explained by a different overall length of path options in the two choice sets. The figure is generated with use of package matplotlib v3.2.2 (https://matplotlib.org/) for Python v3.7.7.

The results of estimating $\beta _j$, the participant-specific coefficients for the cost of walking under the sun, are presented in Fig. 3a. We observe that most participants have an expected value $E[\beta _j] > 1$, indicating depreciation of the sun. Some participants, such as those with codes 31 and 42, have expected values of $\beta _j$ close to 1.8, indicating they perceive walking under the sun as demanding 80% more effort. Figure 3b provides an interpretation of the values of parameter $\beta _j$ through indifference curves. A value of $\beta _j=1$ corresponds to complete indifference between sun exposure and shade, thus walking 100 m under shade is identical to walking 100 m under the sun. As an extreme example, participant 31 had the largest value ($\beta _{31} = 1.84$), which implies that this participant perceived 54 m walked under the sun as demanding as 100 m walked under full shade, or 77 m of mixed exposure (50 m under full shade and 27 m under the sun). The average value of $\bar{\beta }_j=1.16$ (shown in red), indicates that, generally, 100 m walked under full shade is perceived as equal to 86 m under the sun or a combination of 43 m under the sun and 50 under the shade. Indifference curves of individual participants are shown in grey. In Fig. 3a we also observe that the 95% credible intervals are wide, containing values of $\beta _j < 1$. This can be explained by the relatively low number of choices per participant, which do not allow for a more certain estimation of this parameter.

When building the posterior distribution for $\beta _j$ for treatment decisions of all participants (Fig. 3b) we obtain an expected value of $\bar{\beta }_j = 1.16$ at the population level. With this we can conclude that, according to the observed path choices of participants and the proposed model, there is evidence of an additional perceived effort (or cost) of walking under the sun, which is on average equal to 16%. These numbers are different if we pool the decisions per choice set, (Fig. 3d,e) giving a higher expected value of $\beta _j=1.23$ for the choices made under choice set #2. This can be explained by the more stable building shade in choice set #2. This indicates that under certain conditions (i.e. environment facilitating behavioural adaptation) we can expect an even higher estimated perceived cost of walking under the sun. In Fig. 3c–e the credible intervals for $\beta _j$ are wide and span from as low as 0.37 to as high as 2.20. This can be explained by the high variability in the decisions, which cannot always be explained by the sun-shade composition of the path options. In the discussion section we elaborate on the support for $\beta _j < 1$ suggested by the posterior distribution.

The expected value of the perceived tree shade intensity parameter is $E[\rho ]=0.50$ (Fig. 3f). This indicates that tree shade is not considered as intense as building shade. The 95% credible intervals of cost-difference-scaling parameter $\tau _k$ (see “Models” for the full model specification) are disjoint for decisions from the two different choice sets (Fig. 3g), which confirms that our decision to estimate it separately per choice set was necessary.

## Discussion

Heat stress in urban outdoor environments is severely threatened by the changing climate and behavioural adaptation is the only way to thermally regulate in a longer run. Here we applied decision theory and computational modelling to study pedestrian behavioural thermal regulation in semi-controlled natural environments, and in particular, how the participants incorporate sun exposure to reach path decisions. Results confirm the presence of sun avoidance behaviour through path choices. The estimated parameters of the hierarchical probabilistic model of path choices reveal the individual preference of pedestrians towards longer, but shadier paths. Tree shade intensity is considered significantly less intense than building shade, which is reflected in the observed path choices and the estimated parameter of the path choice model.

We find the expected value of the parameter $\beta _j$, reflecting the sun-shade preference of participants, to be $E[\beta _j]=1.16$. This indicates that walking in the sun is considered by pedestrians on average $16\%$ longer as compared to walking the same distance in the shade. Individual participants have exhibited path choices which indicate $E[\beta _j]$ as high as 1.84, an $84\%$ increase in the perceived effort of walking under the sun. This finding confirms that pedestrians actively incorporate the shading of outdoor environments into path choices, demonstrating pronounced thermoregulatory behaviour through sun avoidance. It is important to provide, through smart urban planning and design (e.g. by choosing optimal building height and orientation or by placing dedicated shading infrastructure), the opportunity for such behavioural adaptation to minimize heat stress of pedestrians. Urban spaces designed to accommodate pedestrians and provide more comfortable walking spaces can help promote walking, which in turn can have direct health, economic and environmental benefits^39,40^. For example, our study suggests that, the Walk2Ride programme of Singapore, which provides sheltered pathways in 400 meters radius around main public transportation hubs, makes them on average 14% “closer” perceptually, encouraging more people to walk to subway or bus interchange instead of taking a bus. Our study provides quantitative understanding of the benefits provided by shading in urban context. Quantitatively characterised preferences of people can be integrated into processes of urban planning allowing to estimate the distribution of path and route choices of pedestrians in different scenarios and to perform cost-benefit analyses of potential interventions, such as the one mentioned above.

Our results demonstrate that in the process of path choice, participants differentiate the type of shading. Modelling of the data indicates that tree shade is perceived as less intense than building shade. Incorporating a parameter for this in the model of decisions, we find an expected perceived tree shade intensity $\rho =0.5$, or only half of that associated with building shade. This parameter can vary depending on the type of trees and climate and it may be interesting to compare the objective physical property of tree shade density (e.g. measured by leaf area index^41^ or mean radiant temperature reduction^42^) to the one perceived by pedestrians. Our finding has an important implication for urban planners, suggesting that, while trees are able to provide shading relief, it is not considered as intense as that provided by the built infrastructure and thus has a lower impact on outdoor thermal comfort and a smaller reduction of the associated cost of walking.

An interesting question that arises is whether the strategy adopted by the participants actually translates to minimal accumulation of heat, in which case it would mean that the decision mechanism is another example of a “heuristic”—i.e. simple decision strategies that allow quick, but not necessarily optimal, judgments and decisions. Our study finds that pedestrians often minimise their exposure to the sun by choosing the longer and less sunny path option—yet this could be sub-optimal as it might end up in more heat being accumulated in the body and more thermal discomfort than walking in a hotter but shorter path. Incorporating thermal regulation models^43–45^ into the analysis would allow us to calculate the amount of heat accumulated in the human body when walking on both path options and to determine, what strategy, sun-avoidance or distance-minimisation, results in a lower amount of heat accumulated. Such analyses could also then disclose whether the implied decision mechanisms used are efficient or, on the contrary a heuristic that allows a quick decision which albeit sub-optimal is “good enough” (Herbert Simon’s satisficing criterion^46^). Future research should aim to understand to what extent pedestrian choices reflect decision biases and heuristics as opposed to optimised behaviours.

While every effort was made to control the conditions of the natural outdoor environment, it is inevitable that other personal and environmental factors, such as aesthetic preferences and personal thermoregulatory characteristics, were present during the experiment—and this could explain the “non-optimal” decisions. However, these decisions were incorporated in the final estimates of the parameters of the choice model, making it more robust. In more moderate climates, different, or even opposite, behaviours (i.e. sun preference) can be expected. Conducting similar experiments in other climates with different environmental factors (i.e. aesthetics, visibility or lighting) would provide an opportunity to refine the findings of our study and to contribute further to a comprehensive model of the pedestrian decision process in outdoor environments.

## Methods

### Experimental procedure

The research procedures reported in this paper were carried out in accordance with Swiss Federal Act on Research involving Human Beings and Nanyang Technological University’s policy on Research Involving Human Subjects and were approved by ETH Zurich Ethics Commission (approval no. EK 2018-N-94, 18 January 2019) and by the Institutional Review Board of Nanyang Technological University (reference no. IRB-2019-04-025, 23 May 2019).

This study has been pre-registered prior to data analysis (https://osf.io/q5hnk/).

The experiment was conducted during the period from June to December 2019 in the courtyard of National Institute of Education on the campus of the Nanyang Technological University, Singapore. Students, staff and visitors of the University constitute the sampling population. Participants were recruited through posters, placed on campus, advertising the study. Eligibility requirements listed ages of 21 to 55 years, an overall physical fitness level necessary for walking in outdoor environments and an absence of medical conditions preventing prolonged walking in outdoor spaces.

For each experimental session a 1.5 h time slot was reserved. Participants arrived at a predefined instruction spot located in the outdoor environment, protected from direct sunlight. After studying the information sheet and providing their informed consent, the participant was asked to fill the pre-experiment survey (can be found in pre-registration) containing questions on socio-demographic characteristics of the participant, his/her attitude towards Singapore’s environment and his/her lifestyle. Upon finishing the survey, a physiological wearable sensor (wristband) Empatica E4 was attached to each participant for the purpose of physiological monitoring (data not reported in this paper). The participant was asked to read a short story (for the purpose of recording their baseline physiological signals measured by Empatica E4), after which instructions for the experiment followed. After the participant confirmed his/her readiness, an action camera was put onto her/his chest, to serve the purpose of registering the decisions and the environmental events during the experiment, e.g. start and end of each trial or appearance of the sun.

The participant was directed to the start of the experiment and informed once again about the procedure of the experiment. The participant had to make choices which were given in a choice set booklet (see Appendix A for the choice set booklets given to participants). Trial 0 served the purpose of exploring the environment, in it the participant was asked to walk around the lawn and reach the target. Subsequent trials (trials 1 to 13) were asking participants to reach the target with the paths specified by arrows in the booklet. The target of the previous trial served as the origin of the current one. The participant was asked to visually identify the target and path options in the environment at each decision point. Next, the participant was asked to make decisions based on his/her own preferences, as there was no correct or incorrect choice. The participant was informed, that he/she was not tested for the speed of trial completion. Participants were provided a water bottle to avoid dehydration and were explicitly asked to make use of it at their own discretion. The experimenter has left the participant to complete the trials and was observing the participant from a distance without giving additional instructions. Participants were asked to indicate their need for any help by standing still and raising their hand. Participants, who required intervention of experimenter in their walking trials due to environmental conditions (rain), confusion of paths or other reasons, were dismissed from the analysis reported in this paper. Upon finishing the last trial, the participant was met by the researcher and led back to the instruction location, where sensors were detached. The participant was then asked to fill in a post-participation survey, containing questions on the overall state of the participant, as well as on their motivation for each of the chosen paths, evaluation of climate sensation, perception and acceptance during the trials. After completing the experimental procedures, the participants were debriefed and compensated for their participation with 20 Singapore dollars in cash. Neither recruitment, nor instruction materials included an explicit formulation of the research question of this behavioural study to minimize the bias in their behaviour. Instead, the goal of the study was formulated as follows: ’The goal is to investigate navigational attributes, or features, of outdoor ambulation in a variety of environments within Singapore. In addition we plan to focus on the environment’s influential factors’.

In total 74 participants took part in the experiment. Of them 4 had missing data or could not complete the experiment due to rain, 3 were dismissed due to failing the dummy trial, 9 took unspecified paths or had other navigational problems, which required intervention by the experimenter, 2 participants managed to self-correct their incorrect paths without the experimenter’s intervention, but were still dismissed from the analysis in this study.

### Data processing

The raw datasets resulting from the experiments consist of the video shot on the camera mounted on the participant’s chest, physiological signals originating from the Empatica E4, responses to pre- and post-experimental survey, microclimate data recorded by two Kestrel 5400 portable weather stations installed in the sun and in the shade. In the current paper the data extracted from video recordings was used.

The video-recording of each participant was processed by student research assistants according to a protocol by entering all events from the video into a spreadsheet of a predefined structure. Times on the video, wristband and experimenter’s smartphone were synchronized by matching the synchronization events on the video with camera’s time. The following events were coded by participants:

1. Decision event: start by participant of a particular trial.
2. End of trial event: participant stepping on the target of the current trial.
3. Sun presence event: alteration of sun from one state to another. States are:
  - full sun (sharp shadows are visible on the ground);
  - cloudy sun (soft shadows are visible on the ground);
  - no sun (sun is behind the clouds and no shadows are visible on the ground).
4. Sun exposure event: alteration of exposure to sun from one state to another. States are:
  - No shade (participant walks on the surface exposed to the sun).
  - Tree shade (participant walks on the surface covered by the shadow cast by the tree).
  - Building shade (participant walks on the surface covered by the shadow cast by the building).
5. Water intake event: it appears at recording that participant is drinking water.

For each of the event the following attributes are recorded:

1. Event code;
2. Time of event;
3. XY-coordinates of approximate location of event probed with the mouse click in the realistic model of the space and sun position (described in the next section);
4. For decision events only: indicator of whether option A path was chosen by participant.

All the decisions and end of trial events were cross-coded by two student research assistants and checked for agreement of decision label, sun presence and timing. Data coding disagreements (events disagreeing in decision label, in sun presence or in start or end time by more than 5 seconds) were resolved by a third person (experimenter).

Events data was used in the current study and provided information on decisions made by participants and on the presence of the sun at the moment of decision (determining whether decision is considered as treatment one). Timing information of decision events was used for calculation of the sun-shade composition of the path options by adjusting the sun position in the model described in the following section.

Events diverging from the standard experimental procedure (e.g. intervention of experimenter or participant making a shortcut), or potentially ambiguous events (e.g. uncertainty regarding presence of the sun) were recorded by data coders in the notes file, which was then reviewed by the experimenter and which informed the consequent treatment of the participant’s data (e.g. dismissal from the analysis).

### Calculation of the sun-shade composition of the path options

The 3D model of experimental area was created and imported into a Unity 3D game engine and visually validated for the realistic reproduction of the shading of the walking paths (see Appendix B for a comparison of video shots and reproduction of them in the model).

All the path options were incorporated into the 3D model as the polygons covering the walking surface. As the paths along the building are 6 m wide, they were divided in 5 strips (each 1.2 meters wide). Thus, each path option had 5 polygons (path strips) assigned to it. When calculating the sun-shade composition of the path options at particular trial, the time information from the event files was used to adjust the sun position in the model. Then the rays covering each polygon of a path option (on a grid of 0.1 $\times$ 0.1 m) were shot in a direction towards the sun. The intersection of each ray with tree or building was detected and then the fractions of rays not hitting anything, hitting a tree and hitting a building were considered as the fractions of the sun, tree shade and building shade on a particular path option polygon. The intersection of the rays with the tree were detected as their intersection with the convex hull around the tree crone, thus the tree shades rendered by Unity 3D and those considered in calculation of sun-shade composition of path options may differ slightly. For each path option, the polygon (strip) with the lowest fraction of the sun was considered as representative of the overall sun-shade composition of the path option. Building shade that covered less than 15% (i.e. less than 0.9 m) of the wide paths along the buildings was denoted as insufficient to be considered by the participants and path options with such shading pattern were parameterised as having no building shade.

The length of the path options was calculated as the sum of the lengths of their segments. These were measured with the use of a laser distance meter by two researchers one operating the meter and another holding a mark at which laser was shot. An average of three repeated measurements was taken as a length of path segment. Additionally, the distance from each tree to selected anchor point in the area was measured and 3D tropical trees were placed in corresponding locations in the 3D model. The dimensions of each tree were adjusted to closely match the shading recorded by the chest-mounted action camera during the experiment in two different seasons. See Supplementary materials for comparison of the 3D model with the camera shots.

The length of the sun-lit stretch, tree shade and building shade along the option was calculated as the length of the path multiplied by the fraction of each component (calculation of which is described in the paragraph above).

### Hierarchical model of the choices

We define the following cost function of the path option:

1 $$\begin{aligned} c^{(A)}_{ji} = \beta _{j}[a^{\text {sun}}_{ji} + (1-\rho )a^{\text {tree}}_{ji}]+ a^{\text {shade}}_{ji} + \rho a^{\text {tree}}_{ji}, \end{aligned}$$

where $a^{\text {sun}}_{ji}$, $a^{\text {tree}}_{ji}$ and $a^{\text {shade}}_{ji}$ are the metric distances in the sun, in the tree shade, and in the building shade respectively, of path option *A* of trial *i* presented to participant *j*. $\beta _j > 0$ is the participant specific distance-inflating coefficient (cost factor) of walking under the sun, $\rho \in [0, 1]$ is the parameter of shade intensity (relief) associated with tree shade, common to all the participants. Assuming an equivalent definition for the cost of option *B* ($c^{(B)}_{ji}$), the difference in the option costs is:

2 $$\begin{aligned} \Delta c_{ji} = c^{(A)}_{ji} - c^{(B)}_{ji}. \end{aligned}$$

The probability of choosing path option *A* $p(y_{ji}=1)$ is modelled by logistic function widely used in dichotomous choice models^37^:

3 $$\begin{aligned} p(y_{ji}=1|\Delta c_{ji};\beta _j, \rho , \tau _k) = \frac{1}{1+\exp (\Delta c_{ji}/\tau _k)} \end{aligned}$$

where $\tau _k$ is the cost-difference-scaling coefficient specific to a choice set $k \in \{1, 2\}$.

The hierarchical model of the participant choices described in the Eqs. (1, 2 and 3) has the following prior belief distributions of the model parameters:

4 $$\begin{aligned} {\begin{matrix} &{} d, e \sim \text {Normal}(0, 1) \\ &{} \beta _j \sim \text {Gamma}(\exp [d+e], \exp [d-e]) \\ &{} \tau _k \sim \text {Gamma}(12.5, 50) \\ &{} \rho \sim \text {Beta(1, 1)} \end{matrix}} \end{aligned}$$

Here the chosen way of parameterisation of distribution of $\beta _j$ helps to avoid high correlation in parameters of $\text {Gamma}$ distribution, allowing the NUTS Hamiltonian Monte Carlo sampler to explore the parameter space more efficiently, to prevent divergence and help faster convergence.

The prior for $\tau _k$ is chosen such that $E[\tau _k]=0.2$ – an approximate average down-scaled (by factor of 0.01) length difference between the path options.

**Figure 4:**
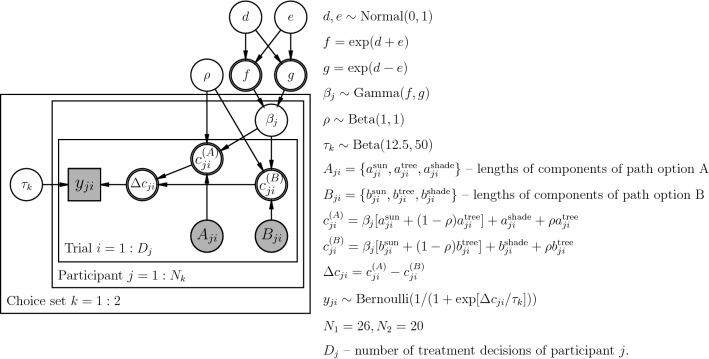
Graphical representation of the hierarchical model of path choices. All variables are continuous except binary $y_{ji}$. Observed variables are shaded, unobserved are not shaded. Of unobserved variables, stochastic ones are single-bordered, deterministic are double-bordered. The figure is generated with use of package daft v0.1.0 (https://docs.daft-pgm.org/en/latest/) for Python v3.7.7.

The full diagram of the model is provided in Fig. 4.

### Markov chain Monte Carlo estimation of the model parameters

We have used the PyMC3^38^ probabilistic programming framework for Python to estimate the parameters of the model. We have used the standard No-U-Turn Sampler^47^, which is based on the principles of Hamiltonian Monte Carlo sampling. The number of chains used is 4, the number of tuning steps is 2000, the number of samples is 10,000 per chain. These parameters achieved a rank-normalized $\hat{R}=1.0$ and effective sample size $>2500$ for all parameters. Thus, there is no indication of lack of convergence of the MCMC sampler.

## Supplementary Information

Supplementary Information.

## Data Availability

The dataset used in the analysis reported in this paper can be found on study’s public OSF page at https://osf.io/aj4vk/.

## Code availability

The Jupyter notebook containing the computer code of analysis reported in this paper can be found on study’s public OSF page at https://osf.io/aj4vk/.
