# Supplementary material for: Behavioural thermal regulation explains pedestrian path choices in hot urban environments
Source: Sci Rep. 2022 Feb 14;12:2441. doi: 10.1038/s41598-022-06383-5 (PMC8844002; doi:10.1038/s41598-022-06383-5)
Supplement: Supplementary file 1 — Supplementary Information. [file 41598_2022_6383_MOESM1_ESM.pdf]

Appendix A. Choice sets

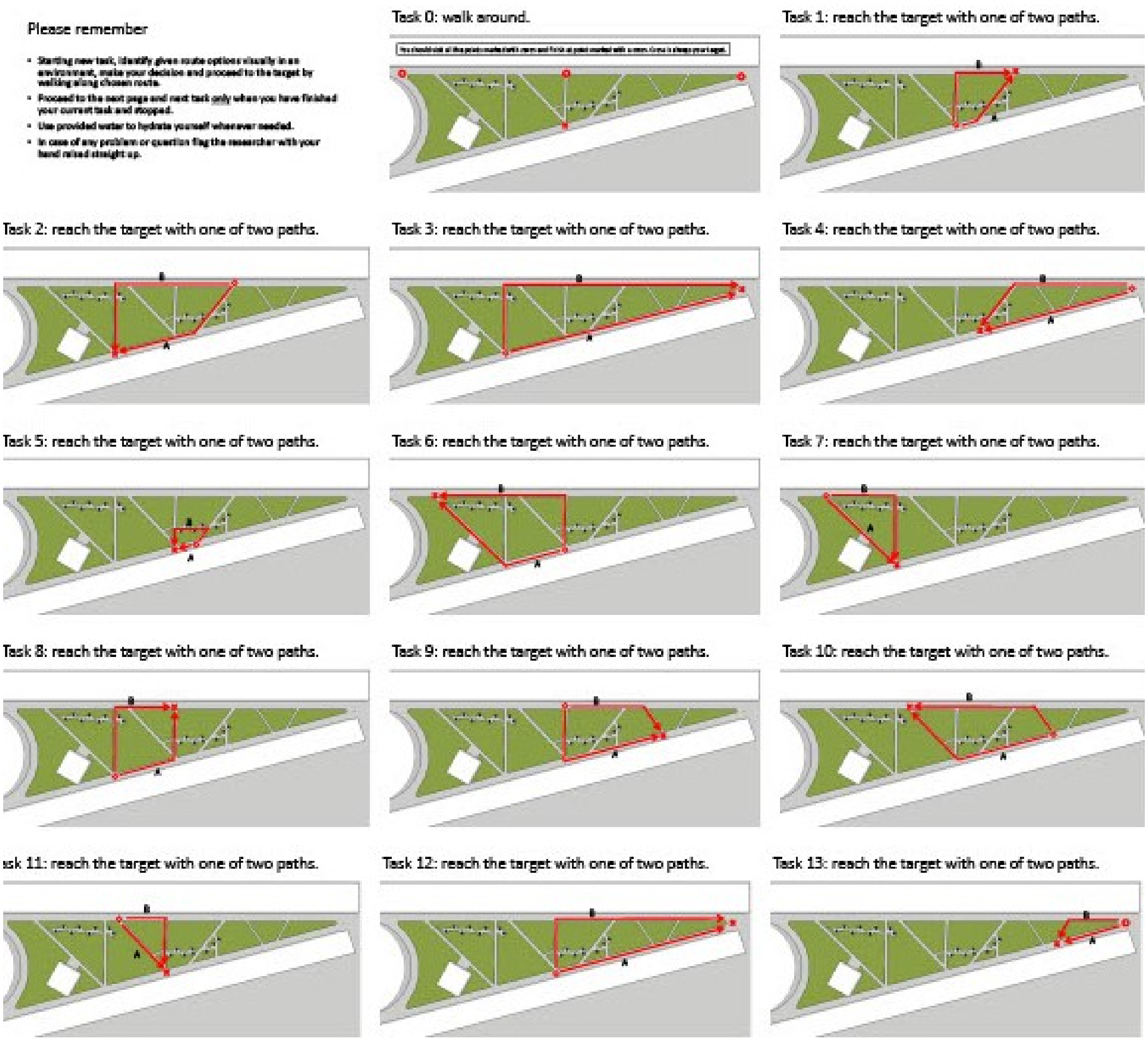

Figure A.1: Pages of the choice set #1 booklet with path options labeled (A and B). These labels were not present in the booklets given to participants.

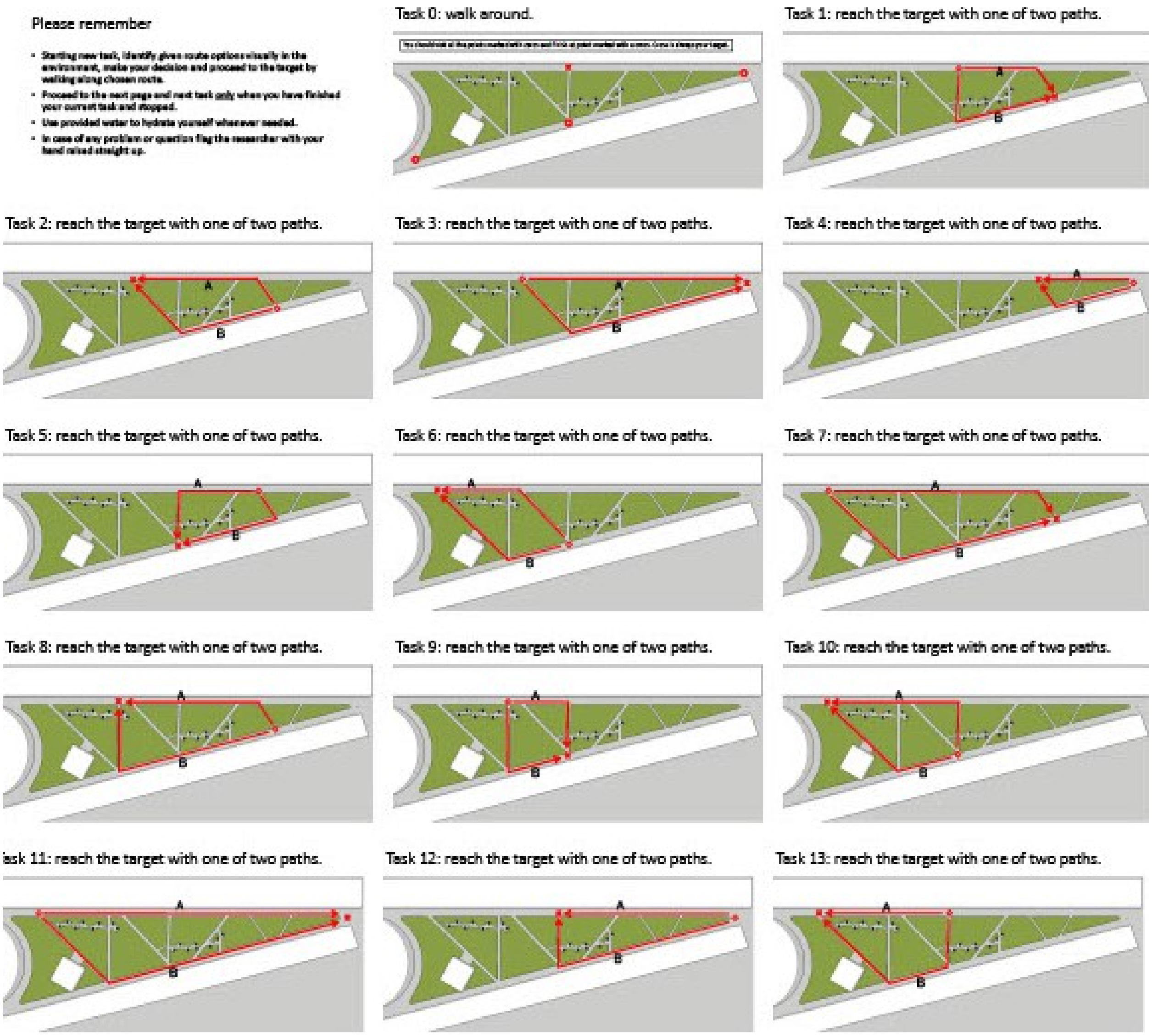

Figure A.2: Pages of the choice set #2 booklet with path options labeled (A and B). These labels were not present in the booklets given to participants.

Appendix B. Demonstration of the accuracy of shading pattern reproduction by the model

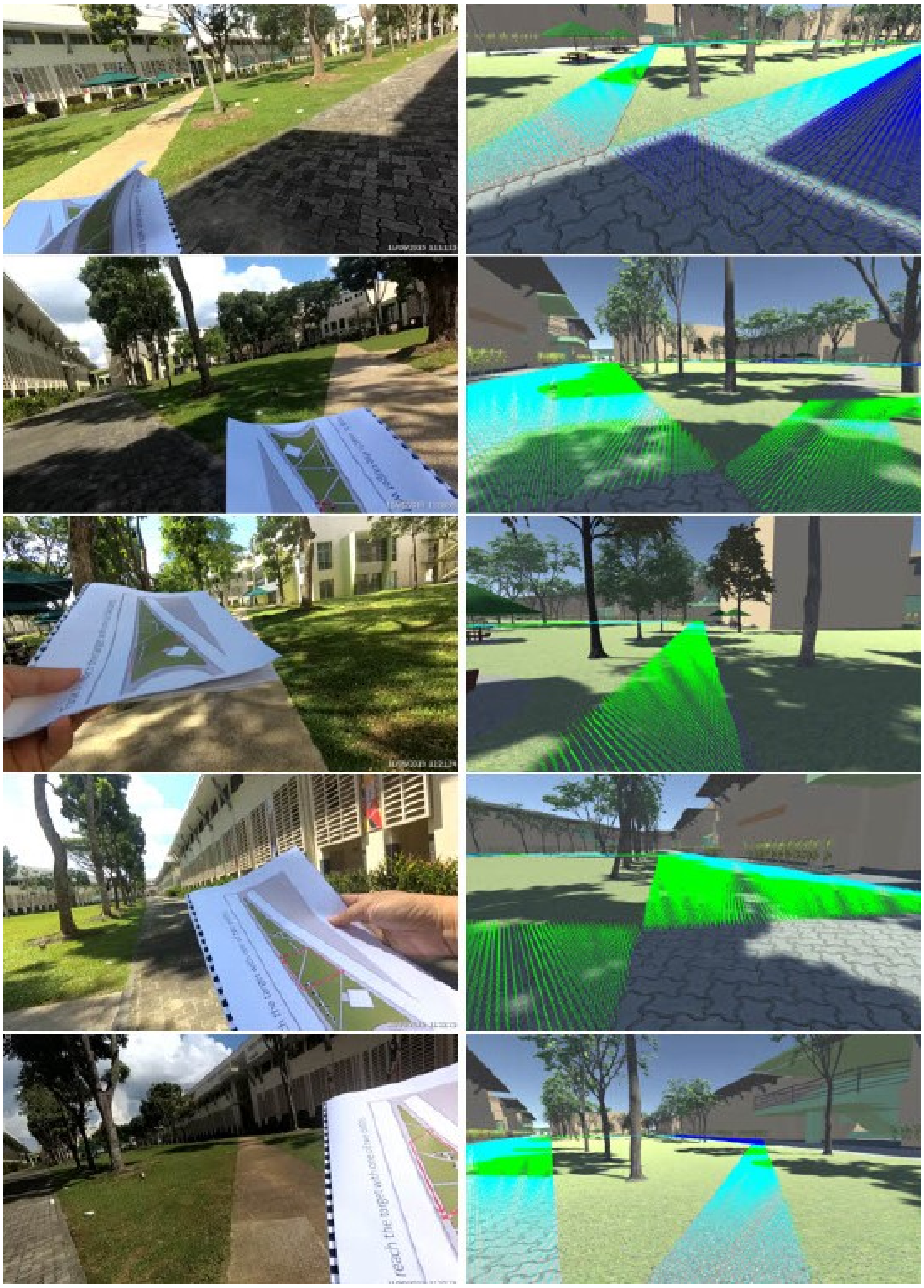

Figure B.3: Comparison of the shading pattern in experimental area on video camera shots and in the 3D model. Participant P02, choice set #1, 11 June 2019, 11:09. Rows from top to bottom depict decision moments of trials: 2, 6, 7, 8 and 10.

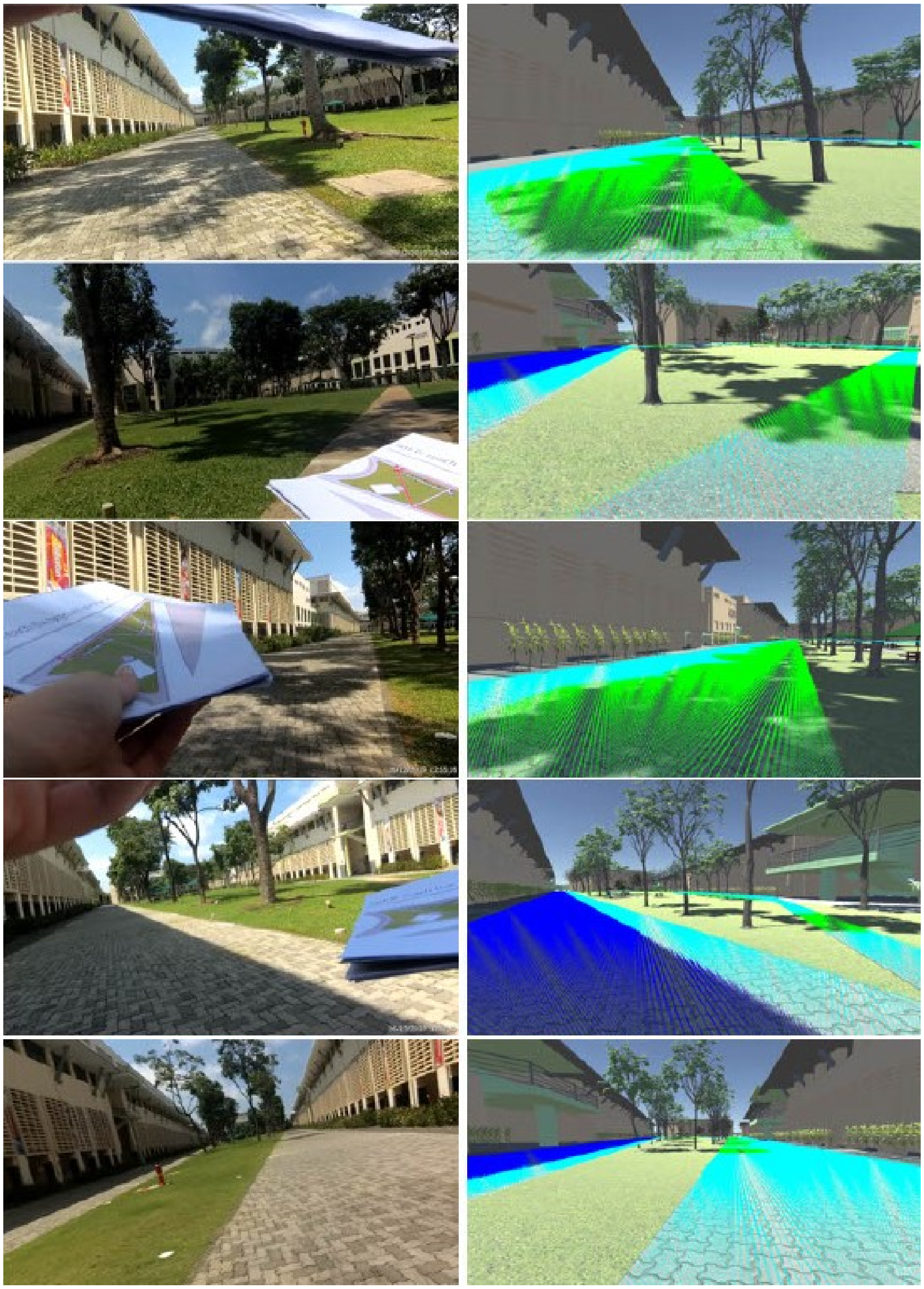

Figure B.4: Comparison of the shading pattern in experimental area on video camera shots and in the 3D model. Participant P45, choice set #2, 26 December 2019, 12:47. Rows from top to bottom depict decision moments of trials: 2, 6, 7, 8 and 12.

Appendix C. Demonstration of importance of perceived tree shade intensity parameter for interpretation of path choices

An example in Figure C.5a demonstrates the sun-shade composition of path options provided to participant 40 in trial 6 of choice set #2. Under the 100% tree shade intensity assumption (tree shade is equal to the building shade) this choice would be 'optimal vs. non-optimal', where a non-optimal decision was made (Figure C.5). Reducing tree shade intensity to 60% results in 40% of the tree shade length of option *A* being considered as full sun (no shade), making the total sun-exposed length of option A longer than that of option B. This results in a change in the choice type, with it now being classified as 'distance-minimising vs. sun-minimising', where a sun-minimising path option was taken.

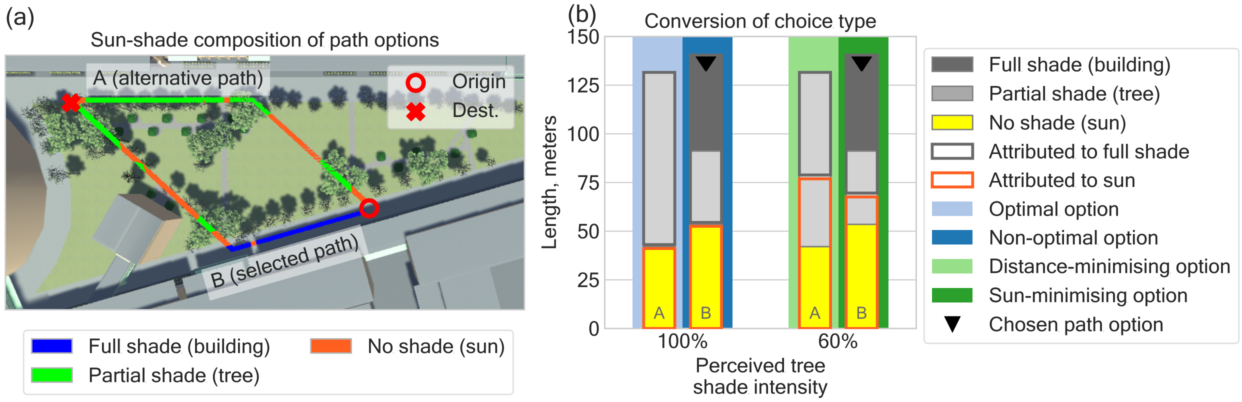

Figure C.5: **Demonstration of a role of a tree shade intensity parameter in classification of choices.** Path choice alternatives and their sun-shade composition in trial 6 of choice set #2 completed by participant 40. (b) Assuming that tree shade is identical to building shade (perceived tree shade intensity is 100%), the choice is between optimal path *A* and non-optimal path B, where the latter was chosen by participant. Assuming that perceived tree shade intensity is 60% (i.e. that 60% of tree shade length is attributed to the full shade and 40% – to the sun) reclassifies the choice into a decision between distance-minimising path A and sun-minimising path B, where the latter was chosen by participant. This figure demonstrates that the perceived tree shade intensity parameter plays a crucial role in the interpretation of the choices of participants and thus was included in our model. The figure is generated with use of package matplotlib v3.2.2 (https://matplotlib.org/) for Python v3.7.7 and Unity 3D (https://unity.com/) v2019.2.19f1.

Figure C.6 shows how the perceived tree shade intensity parameter affects the distribution of decisions of participants by choice type. As the tree shade intensity decreases more choices are classified as 'distance-minimising vs. sun-minimising' (Figure C.6a). The result of this is that many non-optimal decisions are reclassified into sun-minimising decisions (Figure C.6b). The number of sun-minimising and non-optimal decisions is comparable when assuming 100% tree shade intensity (right of Figure C.6b), but as this parameter decreases, the fraction of sun-minimising

decisions increases, suggesting that more than 80% of the longer paths taken in experiment are the result of employment of sun-minimising strategy by participants. In comparison, incorporating the tree-shade intensity results in negligible re-classifications of shorter-path decisions from distance-minimising to optimal C.6c).

The parameterisation of tree shade intensity is to incorporate the possibility that tree shade is perceived as less intense than building shade. This has important implications for the planning of urban areas. To numerically represent this concept, we integrate the tree shade intensity parameter ρ into the choice model.

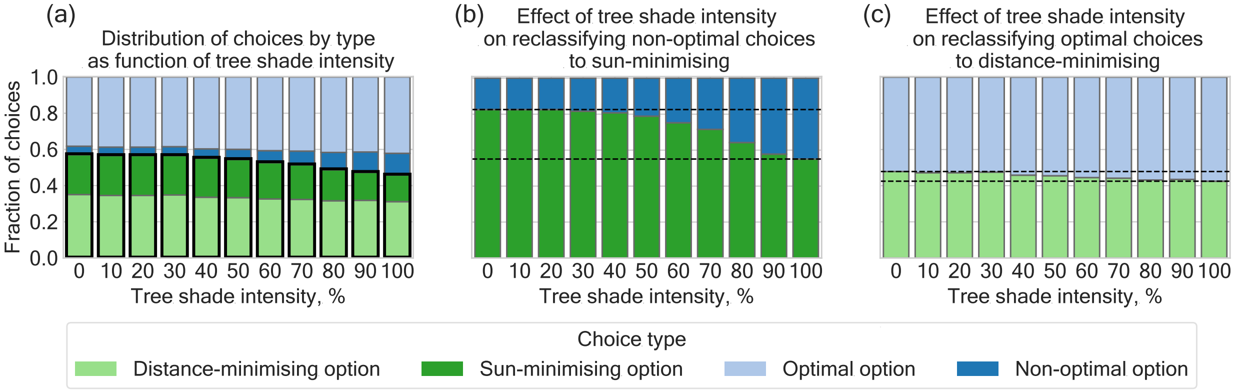

Figure C.6: **Distribution of choices by type as function of tree shade intensity.** (a) As perceived tree shade intensity decreases (read right to left), many choices are re-classified from 'optimal vs. non-optimal' to 'distance-minimising vs. sun-minimising'. Figure (b) shows that the result of this is that a significant fraction of decisions previously classified as non-optimal are now classified as sun-minimising . Whereas, Figure (c) shows that decreasing tree shade intensity results in only a very small fraction of decisions being changed from optimal to distance-minimising decisions. Note that the conversion of sun-minimising decisions into non-optimal (b) only starts at levels of tree shade intensity about 40-50%, providing some evidence for the potential value of this parameter. The exact value of this parameter was formally estimated through modelling the path choices of participants (Figure **??**). The figure is generated with use of package matplotlib v3.2.2 (https://matplotlib.org/) for Python v3.7.7.
